# Profiling Volatile Terpenoids from Calabrian Pine Stands Infested by the Pine Processionary Moth

**DOI:** 10.3390/plants9101362

**Published:** 2020-10-14

**Authors:** Vincenza Foti, Fabrizio Araniti, Francesco Manti, Enrica Alicandri, Angelo Maria Giuffrè, Carmelo Peter Bonsignore, Elvira Castiglione, Agostino Sorgonà, Stefano Covino, Anna Rita Paolacci, Mario Ciaffi, Maurizio Badiani

**Affiliations:** 1 Dipartimento di Agraria, Università Mediterranea di Reggio Calabria, Loc. Feo di Vito, I-89129 Reggio Calabria, Italy; aurorafoti6@gmail.com (V.F.); fabrizio.araniti@unirc.it (F.A.); e.alicandri@gmail.com (E.A.); amgiuffre@unirc.it (A.M.G.); asorgona@unirc.it (A.S.); 2 Dipartimento di Patrimonio, Architettura e Urbanistica, Università Mediterranea di Reggio Calabria, Salita Melissari, I-89124 Reggio Calabria, Italy; francesco.manti@unirc.it (F.M.); cbonsignore@unirc.it (C.P.B.); elvira.castiglione@hotmail.it (E.C.); 3 Dipartimento per la Innovazione nei Sistemi Biologici, Agroalimentari e Forestali, Università della Tuscia, Via S. Camillo De Lellis, s.n.c, I-01100 Viterbo, Italy; stefano.covino80@gmail.com (S.C.); arpaolacci@unitus.it (A.R.P.); ciaffi@unitus.it (M.C.)

**Keywords:** Calabrian pine, *Pinus nigra* subsp. *laricio* (Poiret) Maire, pine processionary moth, *Thaumetopoea pityocampa* (Denis and Schiffermüller, 1775), terpenoids, bornyl acetate, green leaf volatiles, foraging behavior, headspace analysis

## Abstract

Terpenoids make up the biggest and most diversified class of chemical substances discovered in plants, encompassing over 40,000 individual compounds. In conifers, the production of terpenoids, either as oleoresin or emitted as volatile compounds, play an important role in the physical and chemical defence responses against pathogens and herbivores. In the present work, we examined, for the first time to the best of our knowledge, the terpenic defensive relations of Calabrian pine (*Pinus nigra* subsp. *laricio* (Poiret) Maire), facing the attack of the pine processionary moth (*Thaumetopoea pityocampa* (Denis and Schiffermüller, 1775)), brought about in the open on adult plant individuals growing at two distinct forest sites. Among the volatile terpenoids emitted from pine needles, bornyl acetate [(4,7,7-trimethyl-3-bicyclo[2.2.1]heptanyl) acetate] was the most frequently and selectively associated with the infestation, increasing during the period of most intense trophic activity of the caterpillars (defoliation), and decreasing thereafter. Although further work is needed to clarify whether the observed response reflects defence reactions and/or they are involved in communication among the infested plants and their biotic environment, the present results boost the currently growing interest in the isolation and characterization of plant secondary metabolites that can be used to control pests, pathogens, and weeds.

## 1. Introduction

Terpenoids, also referred to as terpenes or isoprenoids, make up the biggest and most diversified class of chemical substances discovered in plants, encompassing over 40,000 individual compounds [1,2,3,4]. The evolutionary success of the terpenoid metabolites largely depends on the flexibility of building molecules of various sizes. Indeed, terpenoids, arising from the two basic five-carbon (C5) isoprenoid units, namely isopentenyl diphosphate and its isomer, dimethylallyl diphosphate, can be categorized as hemiterpenoids (C5), monoterpenoids (C10), sesquiterpenoids (C15), diterpenoids (C20), triterpenoid (C30), tetraterpenoid (C40), or polyterpenoids (C5n), based on the number of C5 units they contain [4,5].

While terpenoids are known to play essential primary functions as precursors of phytohormones and growth regulators, photosynthetic pigments, electron carriers, and key components of membrane structures, “secondary” terpenoid metabolites have been identified as having a range of specialized roles in plant/environment and plant/plant interactions [1,3,6]. Low-molecular-weight terpenoids such as isoprene, monoterpenoids, sesquiterpenoids, and diterpenoids, which are volatile, semi-volatile, or non-volatile at ambient temperature, respectively, are involved in plant defence from abiotic stress and in many above- and below-ground biotic interactions [1,3,7].

The involvement of induced volatile terpenoid compounds in attracting natural enemies of pathogens and herbivores is also well documented (reviewed in [8,9]). Such indirect defence strategy is used by plants to protect their photosynthetic tissues from pathogens and herbivores, as well as to limit insect oviposition [10].

Besides their role in the interaction with herbivores and their antagonists, constitutive and induced volatile terpenoids can act as interspecific, intraspecific, and intraplant signals to promote defence responses in nearby plants or in healthy tissues of the same plant [1,11]. Monoterpenes and sesquiterpenes are particularly suited as long-distance chemical messengers, because of their low molecular weight, high vapor pressure at ordinary temperatures, and lipophilic nature, which facilitates their interactions with membrane systems [1,3,4]. However, there is still a poor understanding of the molecular mechanisms involved in plant-to-environment communication mediated by volatile compounds, and especially so as far as non-model plant species are concerned.

In conifers, the production of terpenoids, either as oleoresin or emitted as volatile compounds, play an important role in the physical and chemical defence responses against pathogens and herbivores [1,12,13,14].

The objective of the present work was to study, for the first time, to the best of our knowledge, the terpenic defensive relations of an endemic conifer of the Calabria territory, namely Calabrian black pine [*Pinus nigra* subsp. *laricio* (Poiret) Maire], facing the attack of the pine processionary moth (PPM) [*Thaumetopoea pityocampa* (Denis and Schiffermüller, 1775)] brought about on adult forest stands in the open.

Currently, black pine covers a large expanse of over 3.5 million hectares [15], making it one of the most widespread conifer species in the Balkans and Asia Minor. Its widest distribution worldwide is in Turkey, with more than 2.5 million hectares [16]. Outside Europe, it has become naturalized in the midwestern states of the US, normally south of the normal ranges of native pines, where it is known as Austrian pine, and also in northern states in New England, around the Great Lakes, and in the Northwest [16]. *Pinus nigra* subsp. *laricio* (Poiret) is one of the six subspecies of black pine; it is found in Corsica and in southern Italy, with a natural range extending from Calabria to Sicily [17]. In Calabria, where it is considered an endemic species, it grows in the Sila and Aspromonte mountains and represents an essential element of the forest landscape, playing an important role not only in soil conservation and watershed protection, but also in the local forest economy [17].

There are about 40 different species of processionary moths, the most widespread in Italy being *Thaumetopoea pityocampa*. This is one of the most important defoliators of pine trees in the Mediterranean region, and its eruptive dynamics inflict serious economic and ecological losses, with desiccation and defoliation, which can even lead to the death of the attacked tree, as well as severe allergic reactions in humans and other mammals [18]. This insect is limited by the low winter temperatures, and the current climate warming is consequently expanding the limits of its distribution to more northerly territories and to higher altitudes, where it was not previously common [19]. *Pinus nigra* is among the preferred species of such insect.

To shed light on the chemical interactions among Calabrian pine and PPM, we aimed mainly to determine whether the concentrations of volatile terpenes differ between infested individuals (I plants in the following) and unattached ones (not infested plants; NI, in the following), from both the qualitative and semi-quantitative point of view, to evaluate which substance(s) might be involved in plant protection. To do this, we measured the volatile terpenoids emitted from pine needles by means of gas chromatography/mass spectrometry (GC/MS) analysis.

To increase its informative and predictive value, the study of plant-host interactions was matched with the biological cycle of the insect, by collecting plant material for analysis throughout almost all of its phenological phases, to understand in which of these the plant might show the need to modulate the emission of its volatile terpenes to possibly implement its defensive strategies. To such aim, samples of Calabrian pine needles were periodically collected at each of two different forest locations, namely Bova and Canolo, within the premises of the Aspromonte National Park, Southern Italy. The first sampling of pine needles was carried out when the young PPM larvae spend most of their time inside their nests and come out sporadically to start feeding (Sampling # 1 - NEST stage, in the following). The second and third samplings were coincident with the maximal trophic activity of the insect, occasionally leading to massive defoliation (Sampling #2 and #3, denoted as DEFO-1 and DEFO-2, respectively, in the following). The fourth and fifth samplings took place at the beginning of the “subsidence” of the insect underground, preceded by the well known “procession” of the PPM caterpillars (Sampling #4 and #5, denoted as PROCE-1 and PROCE-2, respectively, in the following). Finally, the sixth and final sampling was carried out during the stage in which adult PPM females lay their eggs (Sampling #6, denoted as oviposition, OVI, in the following; see Materials and Methods for further details).

An additional aim of the present study was to characterize, for the first time, the blend of volatile terpenes emitted by the studied forest stands under the peculiar environmental conditions occurring in the Aspromonte territory, to learn lessons concerning forest management practices and the possible technological exploitation of these substances.

## 2. Results

Headspace GC-MS analysis revealed twenty-one volatile compounds, all of which monoterpenes or sesquiterpenes, released by the Calabrian pine needles (Table 1).

When a more restrictive criterion was applied, in which only compounds present in at least two replicates out of three were considered, Table 2 and Table 3 were obtained, in which volatiles from NI and I plants are listed in decreasing order on the basis of % area of each peak over the total area of the chromatogram.

**Figure 1:**
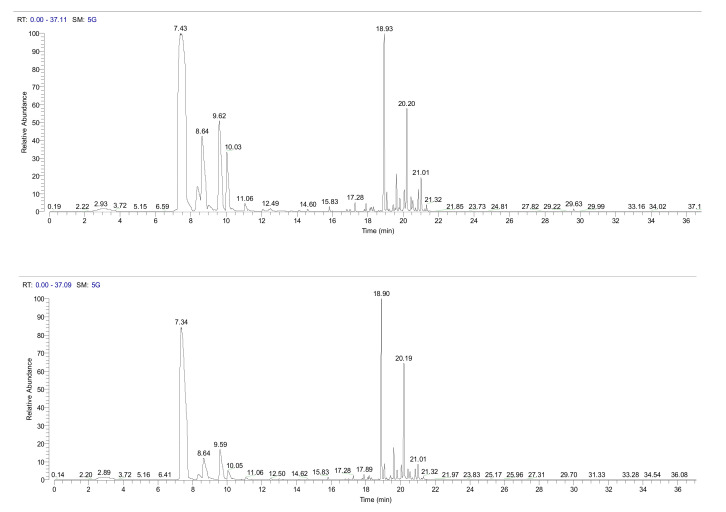
A typical headspace-GC/MS chromatogram obtained from Calabrian pine needles collected at Bova. Upper panel, plant infested by the pine processionary moth; lower panel, not infested (control) plant.

**Figure 2:**
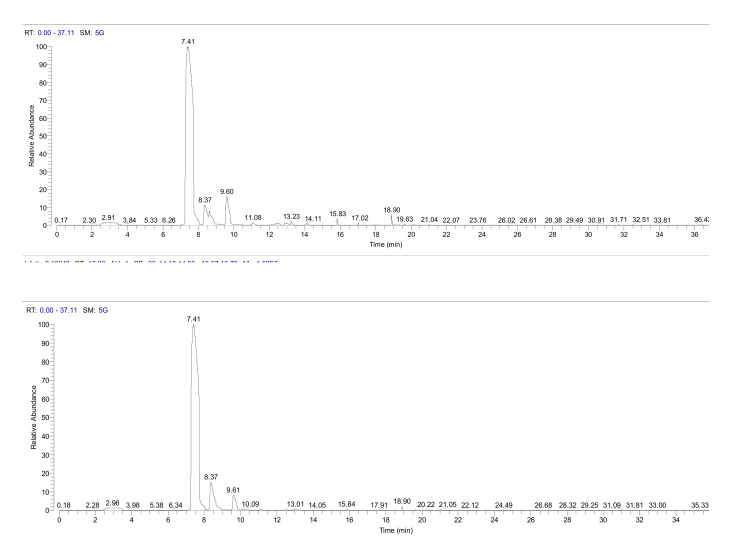
A typical headspace-GC/MS chromatogram obtained from Calabrian pine needles collected at Canolo Nuova. Upper panel, plant infested by the pine processionary moth; lower panel, not infested (control) plant.

Figure 1 and Figure 2 are intended to exemplify that the needles of I plants in Bova seemed to have a more massive terpenes emission than their NI counterparts (Figure 1), but not in Canolo (Figure 2).

**Figure 3:**
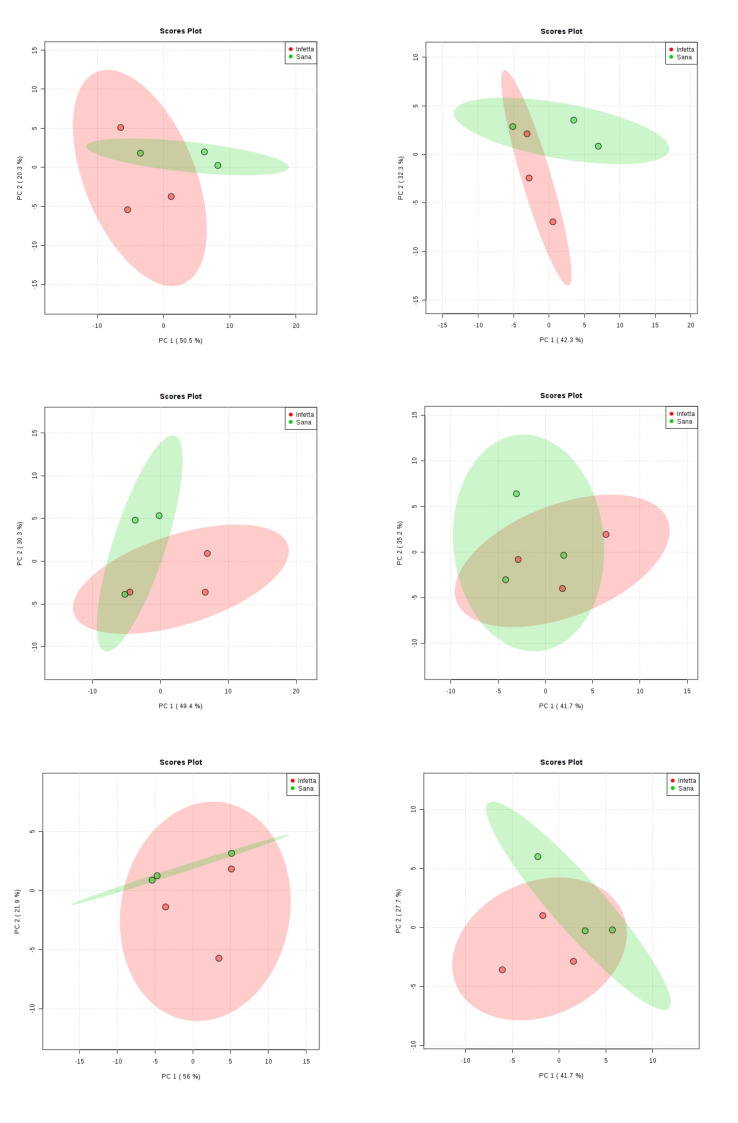

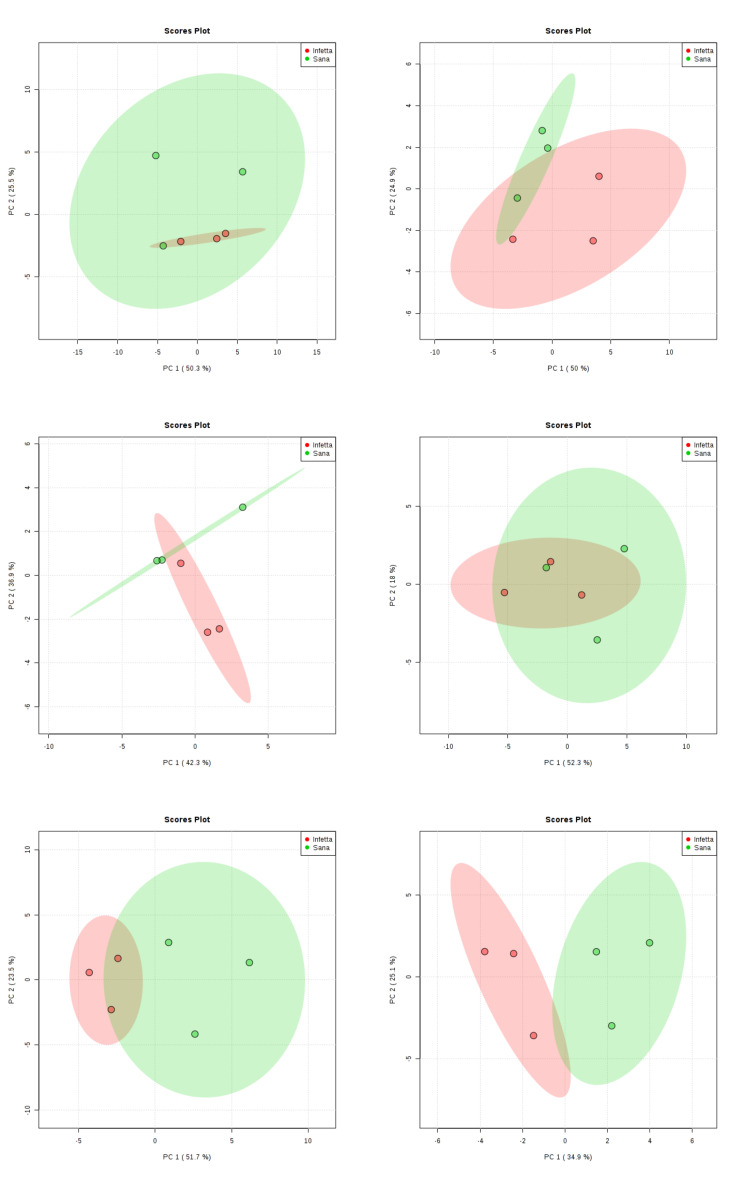
Principal component analysis applied to volatiles emission data obtained from pine processionary moth-infested (red circles) or not infested (green circles) pine needles collected in Bova (left) or in Canolo (right) during six samplings along the insect biological cycle.

Figure 3 shows the results obtained by applying principal component analysis (PCA) to the volatiles dataset from the two sampling locations and from the six sampling times. It is apparent from the results shown that PCA was not able to separate I plants from NI ones, because of the high variability among samples. Therefore, data were further analysed using partial least squares discriminant analysis (PLS-DA)

**Figure 4:**
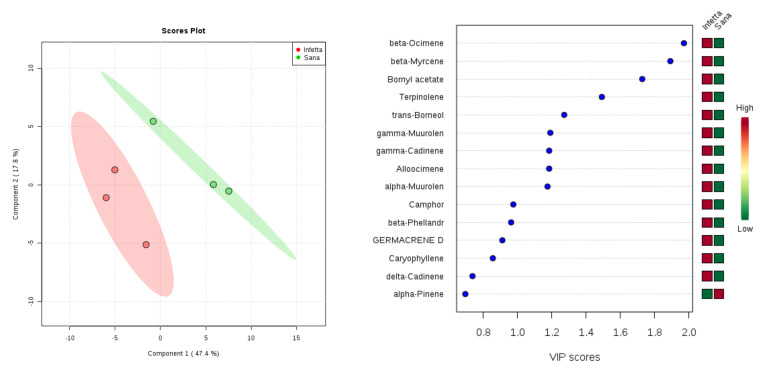
Partial least squares discriminant analysis applied to the volatiles emitted from pine needles collected upon the Sampling #1 (NEST)in Bova. Symbols as in Figure 3.

PLS-DA applied to the NEST sampling (# 1) in Bova (Figure 4) showed that components 1 and 2 alone explained 65.2% of the variability. For NI and I plants, the first two components (1 and 2) helped explain 47.4% and 17.8% of the variability, respectively. In order to identify the volatiles responsible for the discrimination among infested plants and not infested ones, variable importance for prediction (VIP) scores were calculated. Such analysis revealed that the metabolites with the highest VIPS in the NEST samples from Bova were β-ocimene, β-myrcene, bornyl acetate, and terpinolene, all of which were up-accumulated in the PPM-infested plants (Figure 4).

**Figure 5:**
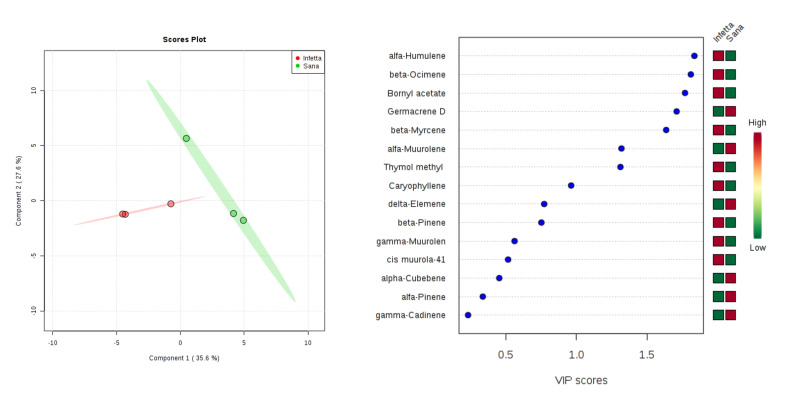
Partial least squares discriminant analysis applied to the Sampling #2 (DEFO-1) in Bova. Parameters and symbols as in Figure 4.

In the DEFO-1 sampling (#2) in Bova (Figure 5), components (latent variables) 1 and 2 explained 63.2% of the variability. In particular, PC1 explained 35.6%, whereas PC2 explained 27.6%. VIPS > 1.4 were found for α-humulene, β-ocimene, bornyl acetate, germacrene D, β-myrcene, α-muurolene, and thymol methyl. The first three metabolites, the fifth, and the seventh were up-accumulated in the I plants, whereas the fourth and the sixth were up-accumulated in the NI ones.

**Figure 6:**
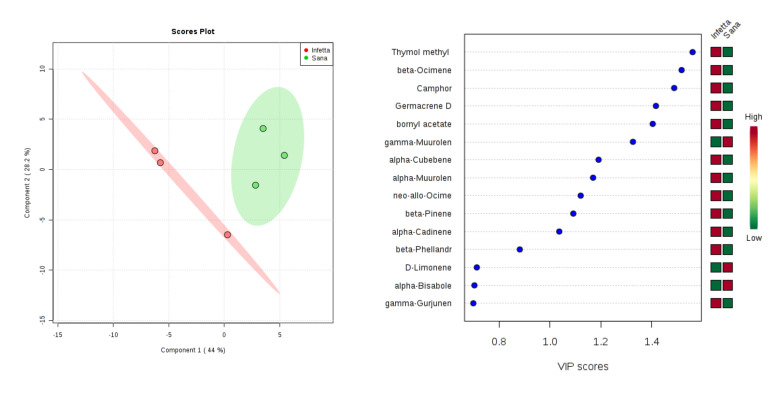
Partial least squares discriminant analysis applied to the Sampling #3 (DEFO-2) in Bova. Parameters and symbols as in Figure 4.

In the DEFO-2 sampling (#3) in Bova (Figure 6), components 1 and 2 explained 72.2% of the variability. In particular, PC1 explained 44%, whereas PC2 explained 28.2%. VIPS > 1.4 were found for thymol methyl, β-ocimene, camphor, germacrene D, and bornyl acetate, all of which were up-accumulated in the PPM-infested plants.

**Figure 7:**
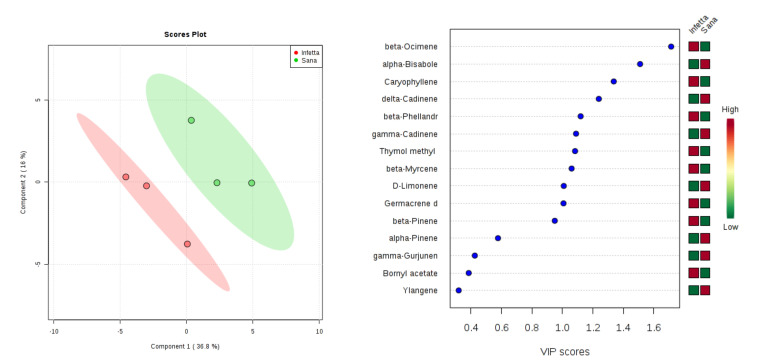
Partial least squares discriminant analysis applied to the Sampling #4 (PROCE-1) in Bova. Parameters and symbols as in Figure 4.

In the PROCE-1 sampling (#4) in Bova (Figure 7), components 1 and 2 explained 52.8% of the variability. In particular, PC1 explained 36.8%, whereas PC2 explained 16%. VIPS > 1.4 were found for β-ocimene, α-bisabolene, and caryophyllene. The first and the third metabolite were up-accumulated in the PPM-infested plants, whereas the second were up-accumulated in the NI plants.

**Figure 8:**
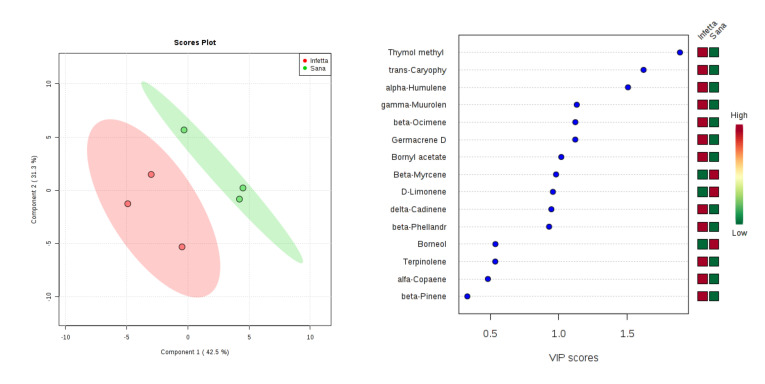
Partial least squares discriminant analysis applied to the Sampling #5 (PROCE-2) in Bova. Parameters and symbols as in Figure 4.

In the PROCE-2 sampling (#5) in Bova (Figure 8), components 1 and 2 explained 73.8% of the variability. In particular, PC1 explained 42.5%, whereas PC2 explained 31.3%. VIPS > 1.4 were found for thymol methyl, *trans*-caryophyllene, and α-humulene, all of which were up-accumulated in the PPM-infested plants.

**Figure 9:**
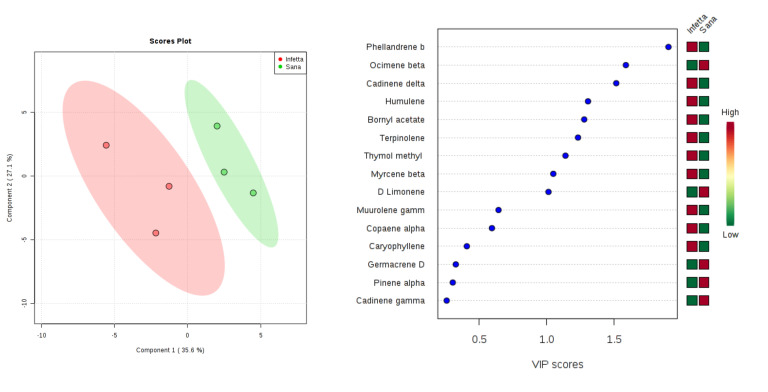
Partial least squares discriminant analysis applied to the Sampling #6 (OVI) in Bova. Parameters and symbols as in Figure 4.

In the OVI sampling (#6) in Bova (Figure 9), components 1 and 2 explained 62.7% of the variability. In particular, PC1 explained 35.6%, whereas PC2 explained 27.1%. VIPS > 1.4 were found for β-phellandrene, β-ocimene, δ-cadinene, humulene, and bornyl acetate. The first, third, fourth, and fifth metabolites were up-accumulated in the PPM-infested plants, whereas the second were up-accumulated in the NI plants.

**Figure 10:**
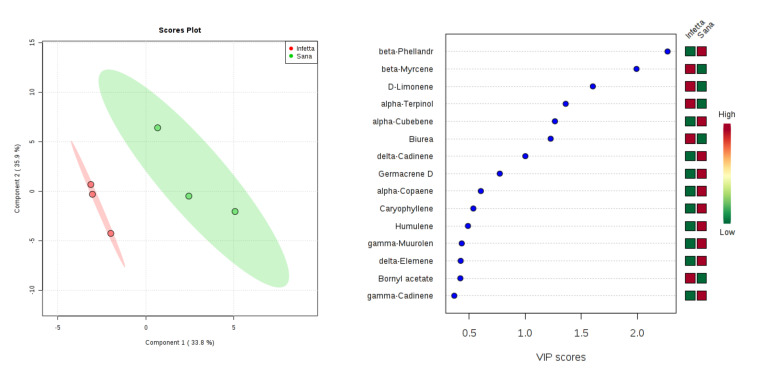
Partial least squares discriminant analysis applied to the Sampling #1 (NEST) in Canolo. Parameters and symbols as in Figure 4.

In the NEST sampling (#1) in Canolo (Figure 10), components 1 and 2 explained 69.7% of the variability. In particular, PC1 explained 33.8%, whereas PC2 explained 35.9%. VIPS > 1.4 were found for β-phellandrene, β-myrcene, D-limonene, and α-terpinolene. The first metabolite was up-accumulated in the NI plants, whereas the remaining three were up-accumulated in the PPM-infested plants.

**Figure 11:**
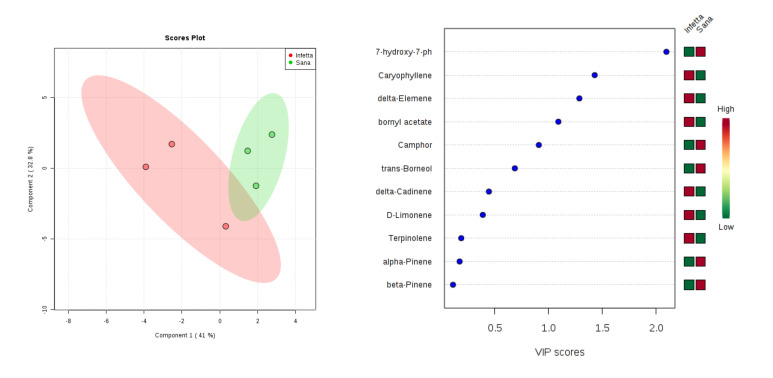
Partial least squares discriminant analysis applied to the Sampling #2 (DEFO-1) in Canolo. Parameters and symbols as in Figure 4.

In the DEFO-1 sampling (#2) in Canolo (Figure 11), components 1 and 2 explained 73.8% of the variability. In particular, PC1 explained 41%, whereas PC2 explained 32.8%. VIPS > 1.4 were found for caryophyllene, δ-elemene, and bornyl acetate, all of which were up-accumulated in the PPM-infested plants.

**Figure 12:**
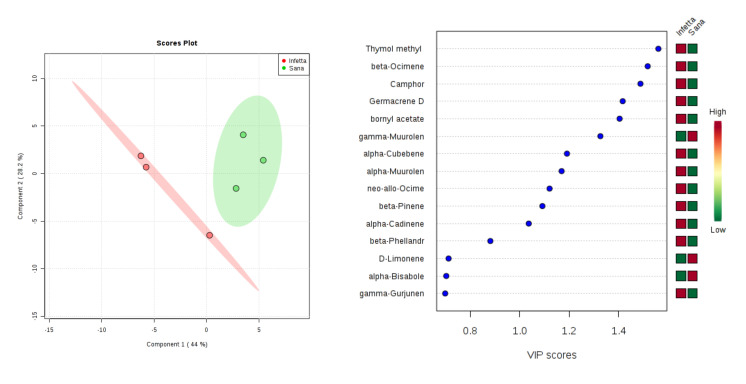
Partial least squares discriminant analysis applied to the Sampling #3 (DEFO-2) in Canolo. Parameters and symbols as in Figure 4.

In the DEFO-2 sampling (#3) in Canolo (Figure 12), components 1 and 2 explained 72.2% of the variability. In particular, PC1 explained 44%, whereas PC2 explained 28.2%. VIPS > 1.4 were found for thymol methyl, β-ocimene, camphor, germacrene D, and bornyl acetate, all of which were up-accumulated in the PPM-infested plants.

**Figure 13:**
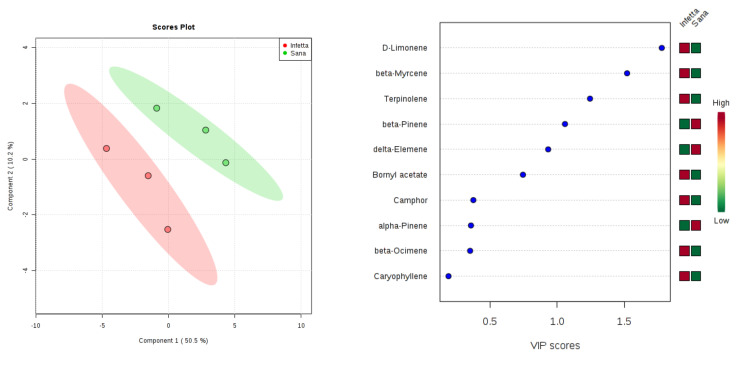
Partial least squares discriminant analysis applied to the Sampling #4 (PROCE-1) in Canolo. Parameters and symbols as in Figure 4.

In the PROCE-1 sampling (#4) in Canolo (Figure 13), components 1 and 2 explained 60.7% of the variability. In particular, PC1 explained 50.5%, whereas PC2 explained 10.2%. VIPS > 1.4 were found for D-limonene, β-myrcene, and terpinolene, all of which were up-accumulated in the PPM-infested plants. 

**Figure 14:**
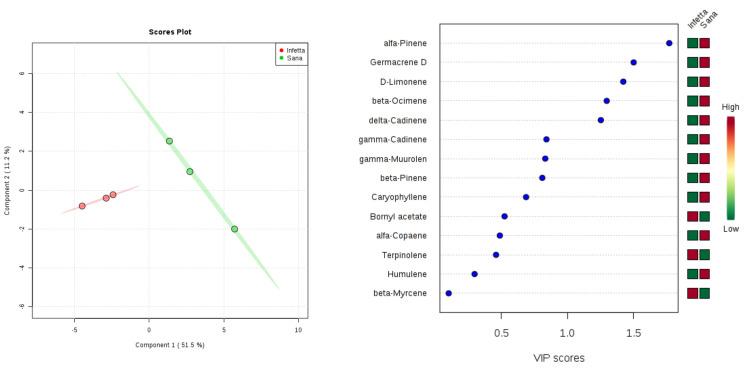
Partial least squares discriminant analysis applied to the Sampling #5 (PROCE-2) in Canolo. Parameters and symbols as in Figure 4.

In the PROCE-2 sampling (#5) in Canolo (Figure 14), components 1 and 2 explained 62.7% of the variability. In particular, PC1 explained 51.5%, whereas PC2 explained 11.2%. VIPS > 1.4 were found for α-pinene, germacrene D, D-limonene, β-ocimene, and δ-cadinene, all of which were up-accumulated in the NI plants.

**Figure 15:**
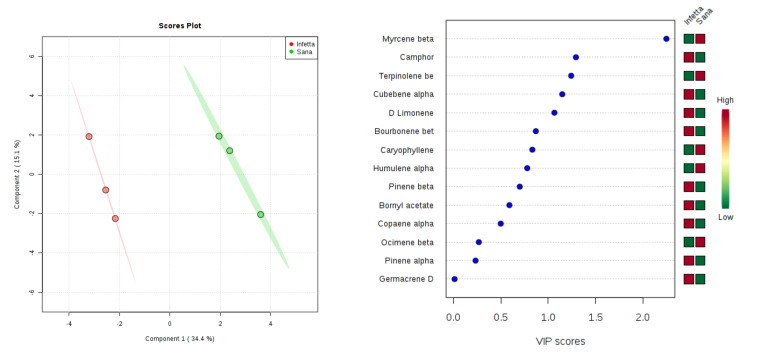
Partial least squares discriminant analysis applied to the Sampling #6 (OVI) in Canolo. Parameters and symbols as in Figure 4.

In the OVI sampling (#6) in Canolo (Figure 15), components 1 and 2 explained 49.5% of the variability. In particular, PC1 explained 34.4%, whereas PC2 explained 15.1%. VIPS > 1.4 were found for β- myrcene, camphor, β-terpinolene, and α-cubebene. The first and the third metabolites were up-accumulated in the NI plants, whereas the second and the fourth were up-accumulated in the PPM-infested plants.

**Figure 16:**
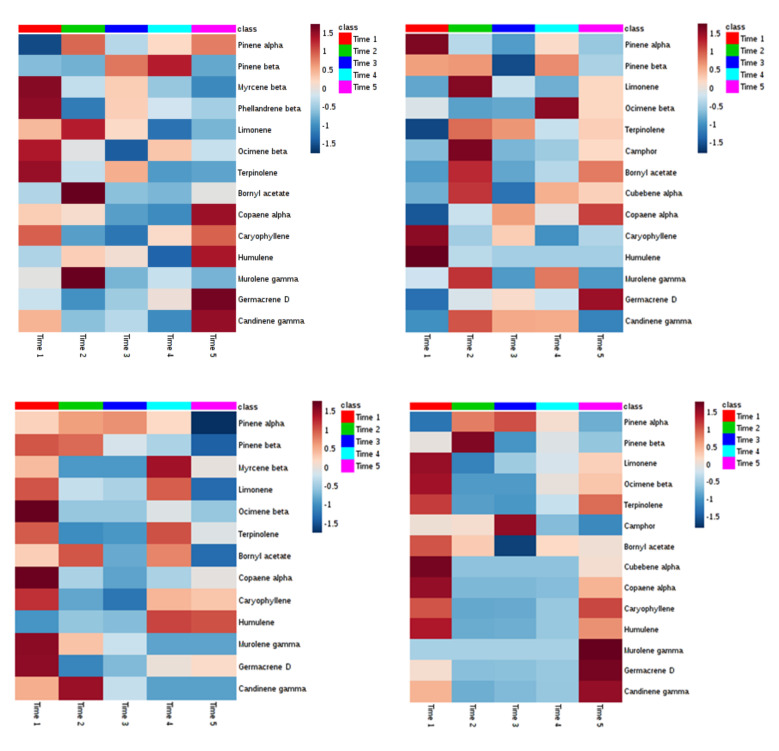
Heatmap synopsis of the changes in the emission of volatile terpenoids from Calabria pine needles (y axis) at five sampling points (x axis) during the trophic season of the pine processionary moth caterpillars. Brownish colors indicate increase, bluish colors indicate decrease. Upper left, infested plants in Bova; upper right, not infested plants in Bova; lower left, infested plants in Canolo; lower right, not infested plants in Canolo.

The four heatmaps shown below (Figure 16), one for each of the two experimental variants and for each of the two sampling sites, aim at presenting a synopsis of the temporal semi-quantitative changes observed in the emission of green leaf volatiles from Calabrian pine needles during the course of the sampling campaigns. 

By considering only those of the aforementioned green leaf volatiles showing the highest VIPS, and by using a more restrictive criterion, the terpenoids shown to be present in all or almost all the headspaces analysed were the two monoterpenes, bornyl acetate and β-ocimene.

The data reported above suggest that, at both sampling sites, a differential emission of bornyl acetate took place from the needles of I plants as compared to their respective NI counterparts, being higher in the former. Such difference was maximal during the period of more intense trophic activity of the PPM larvae, i.e. in DEFO-1 and DEFO-2 samples, and tended to disappear thereafter.

## 3. Discussion

The role of terpenes in insect-conifer interactions has been extensively studied since the 60 s–70 s of the previous century [20,21,22]. A topic attracting most attention has been bark beetle‒conifer interactions, due to the importance of terpenes in these relations, and the great economic and ecological impacts of these pests, especially in the American forests [20,23]. Folivorous insects (insects feeding on conifer needles, such as Lepidoptera) also have important impacts on conifer forests [24].

Plant defences and herbivorous attacks can raise [25] or decrease [26] the concentration of terpenoids and can induce changes in the composition and production of resins and in the emission of volatiles, including terpenes [27]. The emission of volatile terpenes by conifers also has important functions in indirect resistance, because these compounds act as airborne molecular messengers that deter herbivores, attract parasitoids of herbivores [28], or warn other plants of attack, but may also be used as an olfactory cue by herbivores for their host selection [29]. This fascinating complexity makes the role of terpenes in the defence of plants against defoliators controversial, needing further study to understand the functioning of these interactions.

In the present work, analysis of green leaf volatiles emitted from Calabrian pine needles challenged by PPM infestation was carried out both qualitatively and semi-quantitatively. To the best of our knowledge, no previous study of this kind has been carried out concerning such plant-host interaction. Indeed, in a study dating back to the seventies of the previous century, Arbez et al. [30] measured terpenoids in the oleoresins extracted from Calabrian pine, but the analytical approach they adopted was traditional gas-chromatography, i.e., not interfaced with mass-spectrometry; furthermore, no differentiation among volatile and non-volatile terpenoids was attempted, and most importantly, no plant-insect interaction was considered. On the other hand, and much more recently, the role of volatile terpenoids in the arm race among conifers and PPM was studied in detail by Peñuelas and co-workers [31,32], but the plant species involved was Scots pine (*Pinus sylvestris* L.), instead of *P. nigra.*

The results reported here suggest that bornyl acetate and, to a lesser extent, β-ocimene foliar emissions differentiated PPM-infested plants from their respective non-infested controls, being higher in the former during the period of maximal PPM trophic activity. It is noteworthy that these same differences were observed at both sampling sites, namely Bova and Canolo, which are located at the opposite ends of the Aspromonte National Park, i.e., about 40 km apart from each other as the crow flies.

However, such differential terpenoids emission among infested and non-infested individuals was more pronounced in the Bova plants than in the Canolo ones. Speculatively speaking, this might have resulted from the higher degree of PPM infestation observed in the Bova plants, with respect to the Canolo ones, and/or from a different proximity of the PPM nests with respect to the branches chosen for samplings, Canolo trees being larger than Bova ones, thus bearing the PPM nests at higher heights with respect to the sampled branches (see Materials and Methods for further details).

Bornyl acetate is a monoterpene known to be involved in plant defence, and the present study suggests in fact that its emission increased during the period of most intense trophic activity of PPM caterpillars. However, the previous studies available on this topic suggest that the role of such volatile terpenoids in plant-insect interactions might be complex. For example, Cates et al. [33] carried out an agar diet study on Western spruce budworm populations, to determine the effects of varying concentrations of nitrogen, β-pinene, and bornyl acetate on larval growth and survival. Bornyl acetate reduced both growth and survival, suggesting that this compound may be functioning as a toxin or a feeding deterrent. β-pinene, instead, was associated with an increased growth rate and may function as a feeding stimulant. On the other hand, Ryan and Guerin [34] found that (-)-bornyl acetate can act as a host-location cue for the carrot fly larva *Chamaepsila rosae* Fabricius, 1794, and Nishino and Manabe [35] reported that. (+)-bornyl acetate is a mimic of the sex pheromone of the American cockroach (*Periplaneta americana* L.). A recent upsurge of interest for bornyl acetate and other components of essential oils from Lamiaceae, Lauraceae, and Valerianaceae as natural insecticides to combat insects feeding on storage products has come from the studies of Rozman et al. [36] and of Feng et al. [37,38].

The reported results suggest that the presence of defoliator insects influences the emission of specific terpenes by the infested plants. According to the current knowledge, it remains to be ascertained if this might have defensive purposes, either as toxins/repellents towards the attacking insect/attractants for its parasitoids (often referred to as tritrophic interactions),or as an alarm signal to be communicated to the neighboring plants.

## 4. Materials and Methods

### 4.1. Plants Sampling Sites

The Calabrian pine-PPM interaction which is the object of the present work was studied in two artificial and pure pine plantations located near Bova Superiore (Bova, in short) and Canolo Nuova (Canolo, in short), which are located at the southern and the northern limits, respectively, of the Aspromonte National Park, about 40 Km apart from each other as the crow flies, in the southernmost part of continental Italy. Access to the above study areas and sampling of plant material was approved and authorized by the Aspromonte National Park Authority within the framework of an ad hoc research agreement with the Department of Agriculture of the Mediterranean University of Reggio Calabria, Italy.

The sampling area of Canolo (38°33′33′′ N, 16°15′63′′ E; altitude 904 MASL, exposed south), at the northern limit of the park, hosts a main arboreal composition of Calabrian pine and a secondary composition of brushwood with the bracken fern [*Pteridium aquilinum* (L.) Kuhn], with signs of plant renewal of *Quercus* spp. These are mostly adult monoplane high forests, derived from artificial afforestation carried out in the seventies of the previous century. Two Calabrian pine sampling plots were chosen there, one with plants showing visible signs and symptoms of PPM infestation and another representing the not infested control. Three infested plants, each showing several characteristic PPM nests also on branches close to the ground, were chosen. The mean percentage of defoliation due to the PPM caterpillars was around 40%. As many control plants, not showing any visible sign or symptom of PPM infestation, were selected from a nearby forest plot (38°33′92′′ N; 16°14′99′′ E; altitude 950 MASL, exposed south-east), about 100 m apart from the infested plot as the crow flies. Once selected, the Calabrian pine individuals in the two plots were marked with cuttings of white-red signal tape around the trunk bases, in order to facilitate their retrieval. These same marked plants were always used for needles collection throughout the entire sampling campaign (see below).

The sampling area of Bova is located on the eastern slopes of the Aspromonte massif, at the southern limit of the park. Similarly to the Canolo sampling site (see above), Bova also hosts a main arboreal composition of Calabrian pine, which forms mostly adult monoplane high forests derived from artificial afforestation carried out in the fifties of the previous century. As for Canolo (see above), two Calabrian pine sampling plots were chosen at Bova, one with plants showing visible signs and symptoms of PPM infestation and another acting as the not infested control. Three infested plants, each showing several characteristic PPM nests also on branches close to the ground, were chosen from a plot located at 38°1′58′′ N, 15°26′46′′ E, 1194 MASL, exposed south-east. The mean percentage of defoliation due to the PPM caterpillars was around 50%. As many control plants, not showing any visible sign or symptom of PPM infestation, were selected from a plot located at 38°1’58’’ N, 15°26’46" E, 1194 MASL, exposed north-west, about 70 m apart from the infested plot as the crow flies. Once selected, the Calabrian pine individuals in the two plots were marked as described above. These same marked plants were always used for needles collection throughout the entire sampling campaign (see below).

### 4.2. Sampling of Pine Needles

From each of the two sampling areas previously described, and from each infested or not infested plot within each of them, three samples of needles were collected from as many individual plants. Samples were identified as I_1_, I_2_, and I_3_ for PPM-infested plants and as NI_1_, NI_2_, and NI_3_ for not infested (control) plants. Needles were collected by means of a pruner from branches located at 3–4 m from the ground, at 15–25 cm from their tips, taking care to avoid branches showing visible symptoms of defoliation and/or damage, whatever the cause. Once excised from the plant, each collected twig was cut into portions of about 10 cm in length, each bearing one or two tufts of needles, placed inside a transparent plastic bag and stored in a thermal bag at 4 °C. Once back in the laboratory, needles samples were stored (24–48 h) in the fridge at 4 °C until GC/MS analysis.

The number and frequency of pine needles samplings from the aforementioned forest plots was planned a priori by keeping in mind the progression of the PPM biological cycle. A total of six samplings were carried out in each of the two sampling areas: the first sampling (half of February) coincided with the 3rd phenological stage of the PPM, that is, when the young larvae spend most of their time inside the nests and come out sporadically to start feeding on pine needles (denoted as the NEST stage). The second and third samplings (bimonthly during March) were coincident with maximal trophic activity of the insect (4th phenological stage), occasionally leading to massive defoliation (denoted here as the DEFO-1 and the DEFO-2 stages). The fourth and fifth samplings (bimonthly during April) coincided with the 5th phonological stage of the insect, with reduced or no defoliation activity, which marks the beginning of the “subsidence” of the insect underground, preceded by the well known “procession” of the PPM caterpillars (denoted here as the PROCE-1 and PROCE-2 stages). Finally, the sixth and final sampling (late August) was carried at the end of the pupal stage, upon which, after emerging and mating, adult females lay their eggs on the nearest pines (6th phonological stage, oviposition; denoted here as the OVI stage).

Upon each sampling date, plant material was always collected from both the Bova and Canolo experimental sites during the same day, within a total time span of about three hours.

A voucher specimen of the collected plant material has been deposited in the Department of Agriculture of the Mediterranean University of Reggio Caabria, Italy. A total of 2580 g of Calabrian pine needles were collected during the six sampling campaigns

### 4.3. Head Space GC/MS Analysis of Volatiles from Pine Needles

Volatiles from pine needles were chemically characterized by means of Thermo Fisher gas chromatograph apparatus (Trace 1310) equipped with a single quadrupole mass spectrometer (ISQ LT, Thermo Fisher Scientific, Waltham, MA, USA)). The capillary column was a TG-5MS 30 m × 0.25 mm × 0.25 μm; the carrier gas was helium with a flow rate of 1 mL min^−1^. Before the GC/MS analysis, the pine needle samples were taken out from the fridge and left at room temperature for one hour, to allow the emission of volatiles, otherwise inhibited by the low temperatures. Subsequently, needles were selected from the twigs among those not showing evident signs of deterioration or yellowing.

For each sample, one gram of the selected needles was placed inside a SPME incubation vial, the vial closed, and left in a well-balanced position for 90 min. After this time, a SPME device holding a DVB/CAR/PDMS (gray) fiber (Supelco, Bellefonte, PA, USA) was inserted into the vial through its rubber screw cap and left in incubation for 30 min, to allow the sample volatiles to be adsorbed. The fiber was subsequently inserted into the GC injection port, in splitless mode and the volatile substances desorbed.

The gas chromatographic conditions were as follows: isocratic for 3 min at 60 °C, from 60 °C to 240 °C at 6 °C min^−1^, then isocratic for 4 min at 240 °C. The mass spectra were recorded in EI mode at 70 eV, with scanning at 30–300 m/z. The pine volatilome constituents were identified from their retention indices (KI), calculated as relative to the homologous series of (C5–C36) alkanes analysed under the same GC/MS conditions, and by comparison with the built-in mass spectra database of the GC/MS apparatus (NIST 2005 and Wiley 7.0).

### 4.4. Statistical Analysis

The statistical analysis of the volatiles from the Calabrian pine needles was carried out by means of the MetaboAnalyst software, taking into consideration the percentage area of each GC/MS peak. Metabolite concentrations were checked for integrity, and missing values were replaced with a small positive value (the half of the minimum positive number detected in the data). Data were then normalized by a reference sample, by creating a pooled average sample from control groups, transformed through log normalization, to make the metabolite concentration values more comparable among different compounds, and scaled through Pareto scaling, i.e., mean-centered and divided by the square root of standard deviation of each variable [39]. Data were then classified through principal component analysis (PCA). If separation was not achieved, data were further analysed through partial least squares discriminant analysis (PLS-DA), built by using the first two latent variables (or components) allowing sample separation. In order to identify the metabolites responsible for the discrimination among the volatiles profiles, the variable importance for prediction (VIP) score was used to select those with the most significant contribution in a PLS-DA model. VIPs are a weighted sum of PLS weights for each variable and measure the contribution of each predictor variable to the model [40]. Further, the VIP statistic summarizes the importance of the metabolites in differentiating the study groups (i.e., not infested vs infested plants, in the present case) in a multivariate space. In the present experiment, therefore, the volatiles exhibiting the higher VIPS (≥1.4) were assumed to be the most influent variables.

## 5. Conclusions

The present study, which was the first of its kind to be carried out on Calabrian pine, suggests once again how terpenes, despite being considered part of the so-called “secondary metabolism”, are profoundly engaged in all respects in the defence of the plant.

Indeed, and despite a great variability among individual plants living in the open environment, it has been shown here that a specific monoterpene, namely bornyl acetate, is emitted in comparatively greater amounts by plants undergoing pine processionary moth infestation, and especially so during the periods of most intense trophic activity of the insect. Calibrating the sampling of plant material over the biological cycle of the insect, as it was done in the present study, was another remarkable approach of the present research, to obtain a more realistic and reliable scenario of the plant-insect interaction dynamics.

There is no doubt that a better understanding of plant defence mechanisms will be beneficial to agroforestry. More attention will be paid to the study of terpenoids that participate in plant defence responses and the isolation of new secondary metabolites that can be used to control pests, pathogens, and weeds. 

## Figures and Tables

**Table 1:** Volatile organic compounds identified using gas chromatography/mass spectrometry (GC/MS) analysis in the head space of Calabrian pine needles. RT, retention time; KI, retention index.

N.	Common Name	IUPAC Name	Type of Terpene	RT (min)	KI	Structural Formula
1	α-pinene	(1*S*.5*S*)-2.6.6-trimethylbicyclo[3.1.1]hept-2-ene ((−)-α-pinene)	mono	7.38	939	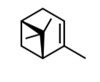
2	β-pinene	6.6-dimethyl-2-methylidenebicyclo[3.1.1]heptane	mono	8.35	982	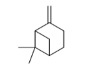
3	β-myrcene	7-methyl-3-methylene-1.6-octadiene	mono	8.75	1000	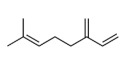
4	β-phellandrene	2-methyl-5-(1-methylethyl)-1.3-cyclohexadiene	mono	9.61	1035	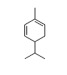
5	limonene	1-methyl-4-(prop-1-en-2-yl)cyclohex-1-ene	mono	9.83	1043	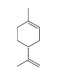
6	β-ocimene	(*Z*)-3.7-dimethyl-1.3.6-octatriene	mono	10.06	1053	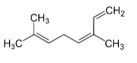
7	terpinolene	4-methyl-1-(1-methylethyl)-1.3-cyclohexadiene	mono	11.09	1095	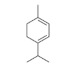
8	thymol methyl ether	2-methoxy-4-methyl-1-propan-2-ylbenzene	mono	11.98	1131	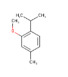
9	camphor	1.7.7-trimethylbicyclo[2.2.1]heptan-2-one	mono	12.48	1152	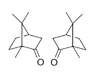
10	bornyl acetate	4.7.7-trimethyl-3-bicyclo[2.2.1]heptanyl) acetate	mono	15.82	1292	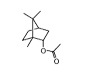
11	γ-gurjunene	(1R.3aR.4R.7R)-1.4-dimethyl-7-prop-1-en-2-yl-1.2.3.3a.4.5.6.7-octahydroazulene	sesqui	16.41	1318	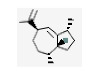
12	δ-elemene	(3 *R*. 4 *R* ) -1-isopropil-4-metil-3- (prop-1-en- 2-il) -4-vinylcyclohex-1-ene	sesqui	16.99	1344	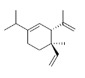
13	α-cubebene	(1R.5S.6R.7S.10R)-10-methyl-4-methylidene-7-(propan-2-yl)tricyclo[4.4.0.0¹.⁵]decane	sesqui	17.27	1357	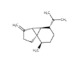
14	α-copaene	(1S.6S.7S.8S)-1.3-dimethyl-8-(propan-2-yl)tricyclo[4.4.0.0^2^.⁷]dec-3-ene	sesqui	17.89	1384	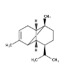
15	β-bourbonene	1-methyl-5-methylidene-8-(propan-2-yl)tricyclo[5.3.0.0^2^.⁶]decane	sesqui	18.82	1427	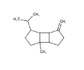
16	caryophyllene	(1*R*.4*E*.9*S*)-4.11.11-trimethyl-8-methylidenebicyclo[7.2.0]undec-4-ene	sesqui	18.88	1430	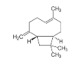
17	α-bisabolene	(*E*)-1-methyl-4-(6-methylhepta-2.5-dien-2-yl)cyclohex-1-ene	sesqui	19.63	1466	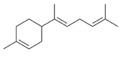
18	humulene	2.6.6.9-tetramethyl-1.4-8-cycloundecatriene	sesqui	19.93	1480	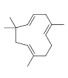
19	γ-muurolene	(1S.4aS.8aR)-7-methyl-4-methylidene-1-propan-2-yl-2.3.4a.5.6.8a-hexahydro-1H-naphthalene	sesqui	20.44	1492	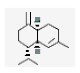
20	germacrene D	(1*E*.5*E*.8*S*)-1.5-dimethyl-8-(prop-1-en-2-yl)cyclodeca-1.5-diene	sesqui	20.18	1502	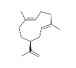
21	γ-cadinene	(1S.4aR.8aR)-7-methyl-4-methylidene-1-propan-2-yl-2.3.4a.5.6.8a-hexahydro-1H-naphthalene	sesqui	20.89	1526	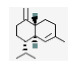

**Table 2:** Volatile organic compounds from the needles of not infested Calabrian pines detected in at least two replicates out of three at each sampling time in the two sampling sites of Canolo and Bova.

Common Name	Type of Terpene	Sampling Time											
		1°-NEST		2°-DEFO_1		3°-DEFO_2		4°-PROCE_1		5°-PROCE_2		6°-OVI	
		Area %		Area %		Area %		Area %		Area %		Area %	
		Canolo	Bova	Canolo	Bova	Canolo	Bova	Canolo	Bova	Canolo	Bova	Canolo	Bova
**α-pinene**	mono	73.043	82.013	89.573	29.990	91.603	75.727	83.950	78.380	76.787	76.630	73.770	77.380
β-pinene	mono	3.106	2.320	4.117	-	2.527	-	3.063	2.403	2.760	2.260	3.980	2.840
limonene	Mono	5.285	4.070	2.410	10.790	2.057	5.680	3.475	4.245	3.935	6.945	4.910	4.990
β-ocimene	mono	4.787	-	-	-	-	-	-	-	3.855	-	6.990	2.620
terpinolene	mono	0.205	-	0.145	0.280	0.140	0.263	0.220	0.193	0.360	0.350	0.810	0.160
camphor	mono	-	-	0.050	-	0.100	-	-	-	-	0.200	-	-
bornyl acetate	mono	-	-	-	0.295	-	-	0.035	0.080	-	0.235	0.130	0.040
α-cubebene	sesqui	0.120	0.195	-	0.265	-	-	-	0.190	-	-	-	0.360
α-copaene	sesqui	0.145	0.135	-	0.445	-	0.190	-	0.180	0.080	-	0.040	0.280
caryophyllene	sesqui	2.780	2.590	-	1.483	0.147	1.903	0.500	1.217	2.867	1.530	2.180	2.700
humulene	sesqui	0.485	-	-	-	-	-	0.055	-	0.330	-	0.160	0.070
γ-muurolene	sesqui		-	-	0.715	-	0.540	-	0.585	-	-	-	0.310
germacrene D	sesqui	0.510	0.830	-	1.690	-	2.120	-	1.550	1.043	2.693	0.100	2.480
γ-cadinene	sesqui	0.500	0.565	-	1.230	-	0.697	-	0.800	0.203	-	0.030	0.670

**Table 3:** Volatile organic compounds from the needles of pine processionary moth-infested Calabrian pines detected in at least two replicates out of three at each sampling time in the two sampling sites of Canolo and Bova.

Common Name	Type of Terpene	Sampling Time											
		1°-NEST		2°-DEFO_1		3°-DEFO_2		4°-PROCE_1		5°-PROCE_2		6°-OVI	
		Area %		Area %		Area %		Area %		Area %		Area %	
		Canolo	Bova	Canolo	Bova	Canolo	Bova	Canolo	Bova	Canolo	Bova	Canolo	Bova
**α-pinene**	mono	1.910	44.673	86.830	66.403	89.867	55.060	72.280	60.040	2.040	65.31	81.517	63.003
β-pinene	mono	74.383	7.505	3.443	4.640	2.607	16.485	3.590	12.930	2.435	6.565	6.643	2.940
β-myrcene	mono	5.060	11.330	-	4.383	-	9.800	8.920	5.040	5.225	-	0.513	3.230
β-phellandrene	mono	-	17.105	-		-	11.890	-	9.960	-	8.785	-	3.460
limonene	mono	10.110	-	-	8.160	3.803	-	9.900	-	-	-	7.530	3.270
β-ocimene	mono	2.080	6.240	-	4.545	-	7.485	-	3.940	-	3.880	0.850	0.190
terpinolene	mono	0.447	0.607	0.205	0.360	0.153	0.470	0.485	0.270	0.283	0.280	0.240	0.310
bornyl acetate	mono	-	0.287	0.270	1.803	0.065	0.133	0.240	0.077	0.100	0.530	0.200	0.380
α-copaene	sesqui	0.060	0.220	-	0.210	-	0.097	-	0.130	-	0.203	0.100	0.220
caryophyllene	sesqui	1.487	4.987	0.467	3.610	0.270	3.350	1.100	4.390	1.050	6.603	1.210	5.610
humulene	sesqui	0.225	-	-	0.387	0.033	-	0.135	-	0.130	0.965	0.100	0.770
γ-muurolene	sesqui	-	0.387	-	0.930	-	0.275	-	0.480	-	0.265	-	0.330
germacrene D	sesqui	0.145	1.137	-	-	-	1.110	-	1.570	-	4.057	0.250	0.410
γ-cadinene	sesqui	-	0.380	-	0.375	-	0.420	-	0.300	-	0.503	-	0.240
